# Clinical utility of the 21-gene assay in predicting response to neoadjuvant endocrine therapy in breast cancer: A systematic review and meta-analysis

**DOI:** 10.1016/j.breast.2021.04.010

**Published:** 2021-05-12

**Authors:** M.G. Davey, É.J. Ryan, M.R. Boland, M.K. Barry, A.J. Lowery, M.J. Kerin

**Affiliations:** The Lambe Institute for Translational Research, National University of Ireland, Galway, Ireland

**Keywords:** Breast cancer, Genomics, Endocrine therapy, Personalised medicine

## Abstract

**Introduction:**

OncotypeDX© Recurrence Score (RS) is a multigene panel used to aid therapeutic decision making in early-stage, estrogen receptor positive (ER+)/human epidermal growth factor receptor-2 negative (HER2-) breast cancer.

**Aim:**

To compare responses to neoadjuvant endocrine therapy (NET) in patients with ER+/HER2-breast cancer following substratification by RS testing.

**Methods:**

This systematic review was performed in accordance to the PRISMA guidelines. Studies evaluating pathological complete response (pCR), partial response (PR), and successful conversion to breast conservation surgery (BCS) rates following NET guided by RS were retrieved. Dichotomous outcomes were reported as odds ratios (ORs) with 95% confidence intervals (CIs) following estimation by Mantel-Haenszel method.

**Results:**

Eight prospective studies involving 691 patients were included. The mean age was 62.6 years (range 25–85) and the mean RS was 14.5 (range 0–68). Patients with RS < 25 (OR: 4.60, 95% CI: 2.53–8.37, *P* < 0.001) and RS < 30 (OR: 3.40, 95% CI: 1.96–5.91, *P* < 0.001) were more likely to achieve PR than their counterparts. NET prescription failed to increase BCS conversion rates for patients with RS < 18 (OR: 0.23, 95% CI: 0.04–1.47, *P* = 0.120) and RS > 30 (OR: 1.27, 95% CI: 0.64–2.49, *P* = 0.490) respectively. Only 22 patients achieved pCR (2.8%) and RS group failed to predict pCR following NET (*P* = 0.850).

**Conclusion:**

Estimations from this analysis indicate that those with low-intermediate RS on core biopsy are four times more likely to respond to NET than those with high-risk RS. Performing RS testing on diagnostic biopsy may be useful in guiding NET prescription.

## Highlights

• Using the 21-gene assay (RS) on core biopsy is useful to guide neoadjuvant therapies.
• Low-intermediate RS are four times as likely to respond to endocrine therapies.
• Neoadjuvant endocrine therapy (NET) failed to increase breast conservation rates.
• Following RS, NET is useful for treating early estrogen receptor positive cancers.

## 1 Introduction

Breast cancer is the most common malignancy in women, carrying a lifetime risk of 12.4% in the western world [1]. In recent years, the prescription of systemic therapies in the preoperative setting has increased rates of breast conservation surgery (BCS) in patients who previously would have been indicated to undergo mastectomy, as well as facilitated axillary down-staging and increasing the proportion of patients spared axillary lymph node dissection, without negatively impacting outcomes [2,3]. Response to systemic chemotherapy is heavily dependent upon the intrinsic tumour properties, with the most favourable pathological complete response (pCR) rates in cancers overexpressing human epidermal growth factor receptor-2 (HER2), or of the triple negative molecular phenotype [4,5]. In clinical practice, patients treated with neoadjuvant chemotherapy (NAC) for estrogen receptor positive (ER+), human epidermal growth factor receptor-2 negative (HER2-) disease typically demonstrate poor response rates [4,5]. Consequentially, NAC prescription in this cohort is typically limited to those with locally advanced (T ≥ 3 and/or N ≥ 2, and M0), stage IIB/III cancers, and those hoping to achieve BCS despite an increased tumour to breast ratio requiring downstaging [6]. A significant proportion of ER + cancers respond favourably to endocrine agents: landmark trials have demonstrated enhanced survival outcomes in ER + disease following administration of endocrine agents [7,8], and current best practice guidelines reflect this as patients with ER + cancers are recommended to receive endocrine therapy for a minimum of five years following surgical resection [9]. Despite knowledge of this sensitivity to such agents, prescription of neoadjuvant endocrine therapy (NET) in ER + disease has been relatively uncommon in clinical practice until recently [10,11].

The molecular era has seen multigene panels, such as the OncotypeDX© Recurrence Score (RS), aid prognostication and therapeutic decision making in patients with ER+/HER2-early breast cancer [12]. Initially, RS testing was applied to select patients believed to derive the most benefit from adjuvant chemotherapy in node negative disease [13,14], although indications are expanding to include locally advanced disease [15]. Observations from Surveillance, Epidemiology, and End Results (SEER) data indicate the majority of ER+/HER2-cancers are low-intermediate risk following RS testing [16], and may be spared chemotherapy prescription [17,18]. Although RS testing is currently used to determine whether adjuvant chemotherapy is beneficial in ER + disease, recent efforts have focused upon the utility of the assay to guide therapeutic decision making in the neoadjuvant setting [19,20].

Recent evidence suggests that RS testing on core biopsy at diagnosis predicts response to NAC in ER+/HER2-breast cancer [19,20]. Specifically, a high RS is associated with increased pCR rates and a low–intermediate RS may indicate relative chemoresistance. Consequently, the performance of RS on core biopsy to guide NAC prescription in patients with ER+/HER2-breast cancer is likely to increase. This suggests that many ER+/HER2-breast cancers will be substratified into less actionable low- and intermediate-risk subgroups that are less likely to benefit from systemic chemotherapy prescription. There is also evidence that NET, even as monotherapy, is associated with similar response rates as NAC within the context of ER+/HER2-disease, but with significantly lower toxicity profile [21].

This paradigm shift has been accelerated by the recent COVID-19 pandemic, as surgeons must balance the risks of a delayed surgery for patients with newly diagnosed breast cancers with the risks of exposure to the virus in this potentially immunocompromised patient cohort, as well as considering the requirement to conserve scarce hospital resources; effectively diverting urgent surgical care to manage a more immediate crisis [22,23]. Studies of tamoxifen with/without surgery demonstrate no difference in survival within the first three years suggesting that short-term deferment of surgery with NET should not adversely impact breast cancer-specific survival [24,25]. Consequently, many expert and organisational guidelines are recommending NET as a ‘bridge to surgery’ for primary resectable ER+/HER2-breast cancer patients during the pandemic, leading to a deviation from the traditional standard of care ‘upfront surgery’ and advising consideration of NET as opposed to NAC for locally advanced ER+/HER2-disease [[26], [27], [28], [29]].

Given these developments, it is a pertinent time to revisit the data surrounding NET and its indications for ER+/HER2-breast cancer. The hypothesis of the current study is that RS may determine patients more likely to respond to, and achieve BCS following NET. Accordingly, the aim of the this analysis was to perform a systematic review and meta-analysis to compare responses to NET in patients with ER+/HER2-breast cancer following stratification by RS testing.

## 2 Methods

This systematic review and meta-analysis was performed in accordance to the Preferred Reporting Items for Systematic Reviews and Meta-Analyses (PRISMA) and MOOSE guidelines [30,31]. Each author contributed to formulating the study protocol and it was then registered with the International Prospective Register of Systematic Reviews (PROSPERO): CRD42020181501.

### 2.1 PICO

Using the PICO framework, the aspects the authors wished to address were:

Population – Female patients with newly diagnosed ER + breast cancer aged 18 years or older without distant metastatic disease who received neoadjuvant endocrine therapy following pre-operative RS testing performed on their diagnostic core tissue biopsy.

Intervention – Any patient in the selected group found to have low (vs. intermediate-to-high) or low-to-intermediate (vs. high) RS on their biopsy.

Comparison – Any patient in the selected group found to have intermediate-to-high (vs. low) or high (vs. low-to-intermediate) RS on their biopsy.

Outcomes – Primary outcomes included: (1) pCR, defined as absence of residual tumour cells in resected specimens following neoadjuvant endocrine therapy (breast and/or axillary pCR); (2) partial response (PR), defined as reduction in degree of residual tumour cells in resected specimens following NET (i.e.: residual cancer burden, Miller-Payne grade, Sataloff classification, etc. As appropriate [[32], [33], [34]]) or (3) successful conversion to BCS following completion of NET.

Secondary outcomes included: (1) disease progression (DP), defined as increased tumour size on clinical or radiological examination following NET; (2) stable disease (SD), defined as no change in tumour size on clinical examination following completion of NET; or (3) 5-year disease-free survival (DFS), defined as freedom from disease recurrence or death at 60 months follow up following cancer resection.

### 2.2 Inclusion and exclusion criteria

Studies included were clinical studies of a prospective nature, including randomised controlled trials, comparing the rates of pCR, PR or BCS, as well as DP, SD or 5-year DFS following NET in patients with low-intermediate versus high RS (or low versus intermediate-high RS) on core diagnostic biopsy. All studies included female patients aged 18 years or greater diagnosed with ER+ (defined in accordance to the American Society of Clinical Oncology/College of American Pathologists as >1% ER expression on immunohistochemical analysis) and HER2- (defined as a score of 0 or 1+ on immunohistochemical staining or HER2-following fluorescence in-situ hybridisation) breast cancer on core tissue biopsy. Published abstracts from conference proceedings were included. Studies including patients with stage four metastatic disease were excluded, as were case reports, case series reporting outcomes in five patients or less, and editorial articles.

### 2.3Search strategy

An electronic search was performed of the *PubMed Medline, EMBASE and Scopus* databases for relevant studies. The final search was performed on the February 6, 2021. This search was performed by two independent reviewers, using a predetermined search strategy that was designed by the senior authors. This search included the search terms: (Oncotype) AND (Neoadjuvant), with AND as a Boolean operator. Included studies were limited to the English language and were not restricted by year of publication. All duplicate studies were manually removed, before titles were screened, and studies considered appropriate had their abstracts and/or full text reviewed. Retrieved studies were reviewed to ensure inclusion criteria were met for one primary and secondary outcome at a minimum. In cases of discrepancies of opinion a third author was asked to arbitrate.

### 2.4 Data extraction and quality assessment

The following data was extracted and collated from retrieved studies meeting inclusion criteria: (1) First author name, (2) year of publication, (3) study design, (4) country of origin, (5) number of patients, (6) neoadjuvant endocrine agent prescribed, (7) duration of neoadjuvant endocrine agent prescription, (8) median age (and range) at diagnosis, (9) RS categorization, (10) clinicopathological and immunohistochemical data (i.e.: menopausal status, tumour stage, tumour grade, progesterone (PgR) status, Ki-67 proliferation indices, etc.) from core biopsy, (11) response rates to therapy (i.e.: pCR, PR, SD, PD), (12) BCS rates, and (13) 5-year DFS rates. Adjuvant chemotherapy prescription was also recorded. Risk of bias and methodology quality assessment was performed in accordance to the Newcastle-Ottawa Scale [35].

### 2.5 Statistical analysis

Descriptive statistics were used to determine associations between RS categories and primary and secondary outcomes. Dichotomous or binary outcome data, reported as odds ratios (ORs) were expressed with 95% confidence intervals (CIs) following estimation using the Mantel-Haenszel method. Either fixed or random effects models were applied on the basis of whether significant heterogeneity (*I*^*2*^ >50%) existed between studies included in the analysis. Symmetry funnel plots were used to assess publication bias. Statistical heterogeneity was determined using *I*^*2*^ statistics. All tests of significance were two-tailed with P < 0.050 indicating statistical significance. Descriptive statistics were performed using the Statistical Package for Social Sciences (SPSS) version 26 (International Business Machines Corporation, Armonk, New York). Meta-analysis was performed using Review Manager (RevMan), Version 5.4 (Nordic Cochrane Centre, Copenhagen, Denmark).

## 3 Results

### 3.1 Literature search

**Fig. 1:**
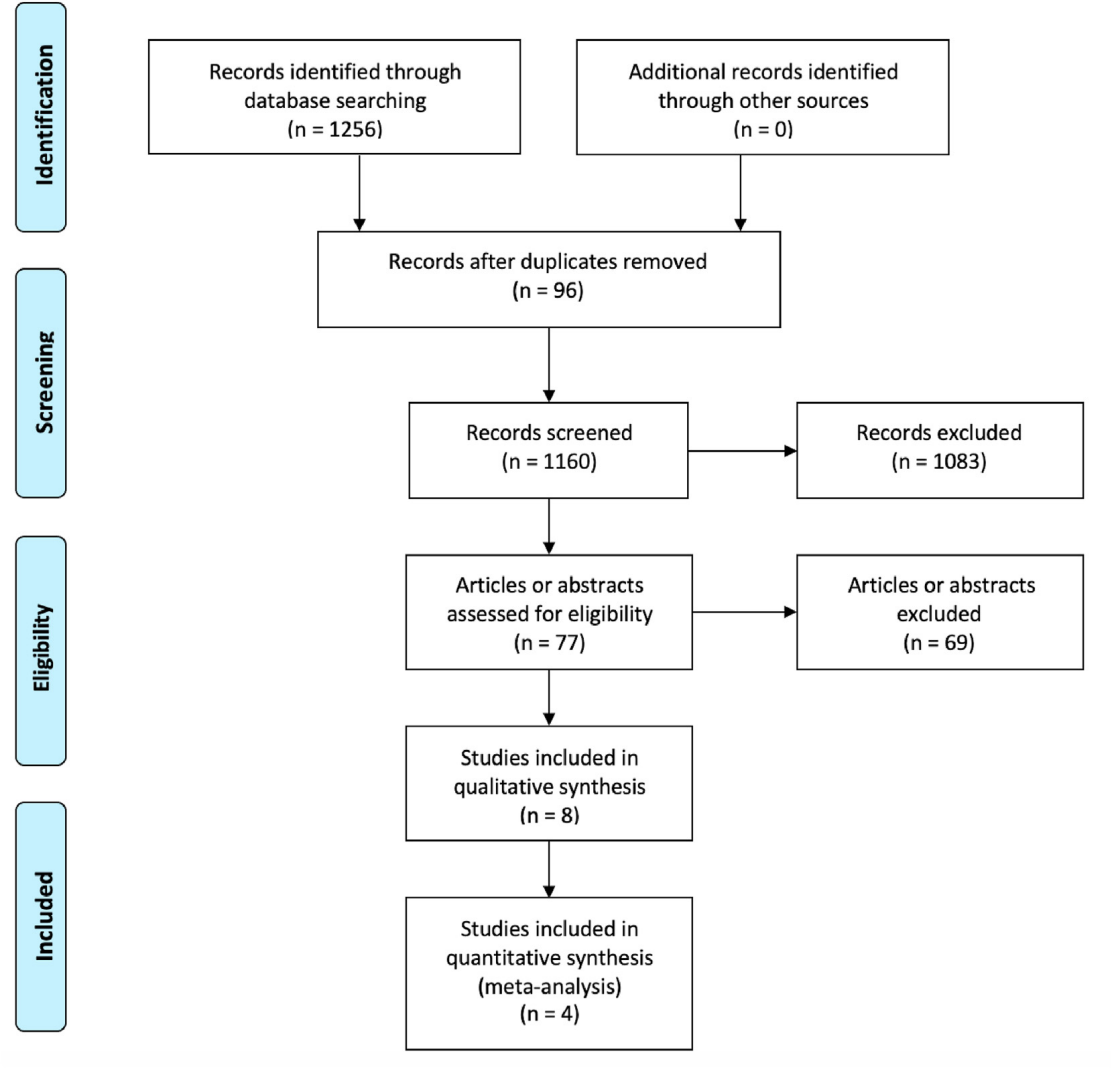
PRISMA flow diagram detailing the systematic search process.

The initial electronic literature search retrieved 1256 studies. Following removal of 96 duplicate studies, the remaining 1160 titles were screened for relevance, before 77 studies had their abstracts and/or full texts reviewed. In total, eight prospective studies fulfilled our inclusion criteria and were included in quantitative analysis, while only four studies were included in meta-analyses due to varying RS cutoffs and data provided (Table 1 & Fig. 1).

**Table 1:** Details of included studies in this analysis.

Author	Year	Study Type	N	NET Agent	NET Duration	RS Cut-Off Value		NOS
						Low	High	
Abu-Khalaf	2019	Prospective	15	Exemestane & AIs	26 weeks	<25	>25	4
Akashi-Tanaka	2009	Prospective	43	Tamoxifen & AIs	16 weeks	<18	>30	7
Al-Saleh	2020	Prospective	238	Fulvestrant & Goserlin	16 weeks	<11	>25	5
Bear	2017	RCT	31	Tamoxifen & AIs	16–26 weeks	<11	>25	6
Iwata	2018	Prospective	295	Letrozole	24–26 weeks	<18	>30	7
Khan	2015	Prospective	42	Fulvestrant & Anastrozole	16 weeks	<25	>25	5
Ueno	2013	Prospective	64	Exemestane	16–24 weeks	<18	>30	7
Ueno	2019	Prospective	59	Exemestane	16 weeks	<18	>30	7

N; Number, NET; Neoadjuvant Endocrine Therapy, RS; OncotypeDX© Recurrence Score, RCT; Randomised controlled trial, AI; Aromatase Inhibitor, NOS; Newcastle-Ottawa Scale.

### 3.2 Study characteristics

Eight prospective studies were included in this study [20,[36], [37], [38], [39], [40], [41], [42]]. Overall, six different endocrine agents (Fulvestrant, Anastrozole, Exemestane, Letrozole, Goserelin & Tamoxifen) were prescribed in the neoadjuvant setting, with duration of treatment varying from 16 to 28 weeks (Table 1). The mean age at diagnosis was 62.6 years (range 25–85) and 78.4% of patients were post-menopausal at diagnosis (501/639–5 studies). The mean RS was 14.5 (range 0–68). Four studies used the traditional numerical categorization of RS: This considered RS < 18 as low-risk, RS 18–30 as intermediate-risk and RS > 30 as high-risk. Four studies used the numerical categorization used in the TAILORx study [17], with RS < 11 considered as low-risk, RS 11–25 as intermediate-risk, and RS > 25 as high-risk (Table 1).

### 3.3 Pre-treatment tumour characteristics

Overall, 691 patients were included in this analysis. RS was low in 257 (37.2%), intermediate in 252 (36.5%), and high in 115 patients (16.6%) (Table 2). The mean pre-treatment tumour size was 28 mm (range: 25–35 mm – 3 studies). Pre-treatment staging was reported in 5 studies: 8.9% were T1 (44/492), 86.8% were T2 (427/492) and 4.3% were T3 (21/492), while 56.8% (79/139) were N0, 31.7% (44/139) were N1 and 11.5% (16/139) were N2 (4 studies). Further clinicopathological and immunohistochemical data retrieved from included studies is outlined in Supplementary Appendix 1.

**Table 2:** OncotypeDX© Recurrence Score broken down by category.

RS Category	Abu-Khalaf 2019	Akashi-Tanaka 2009	Al-Saleh 2020	Bear 2017	Iwata 2018	Khan 2015	Ueno 2013	Ueno 2019	Total	%
Low/Intermediate	15	27	124	31	241	42	49	47	576	83.4%
Low	–	11	23	13	157	–	32	31	257	37.2%
Intermediate	–	16	101	18	84	–	17	16	252	36.5%
High	–	16	18	–	54	–	15	12	115	16.6%
Total	15	43	238	31	295	42	64	59	691	100.0%

RS; Recurrence Score.

### 3.4 Treatment characteristics

Overall, there were 6 different NET regimens prescribed in the 8 included studies (Table 1). Five of the included studies treated patients with dual therapies, and 3 prescribed NET as a monotherapy. NET was prescribed for a mean duration of 20.0 months (±standard deviation 4.2 months, range: 16.0–26.0 months). Table 1 outlines NET regimens for patients included in this analysis.

### 3.5 Response to neoadjuvant endocrine therapy

**Fig. 2:**
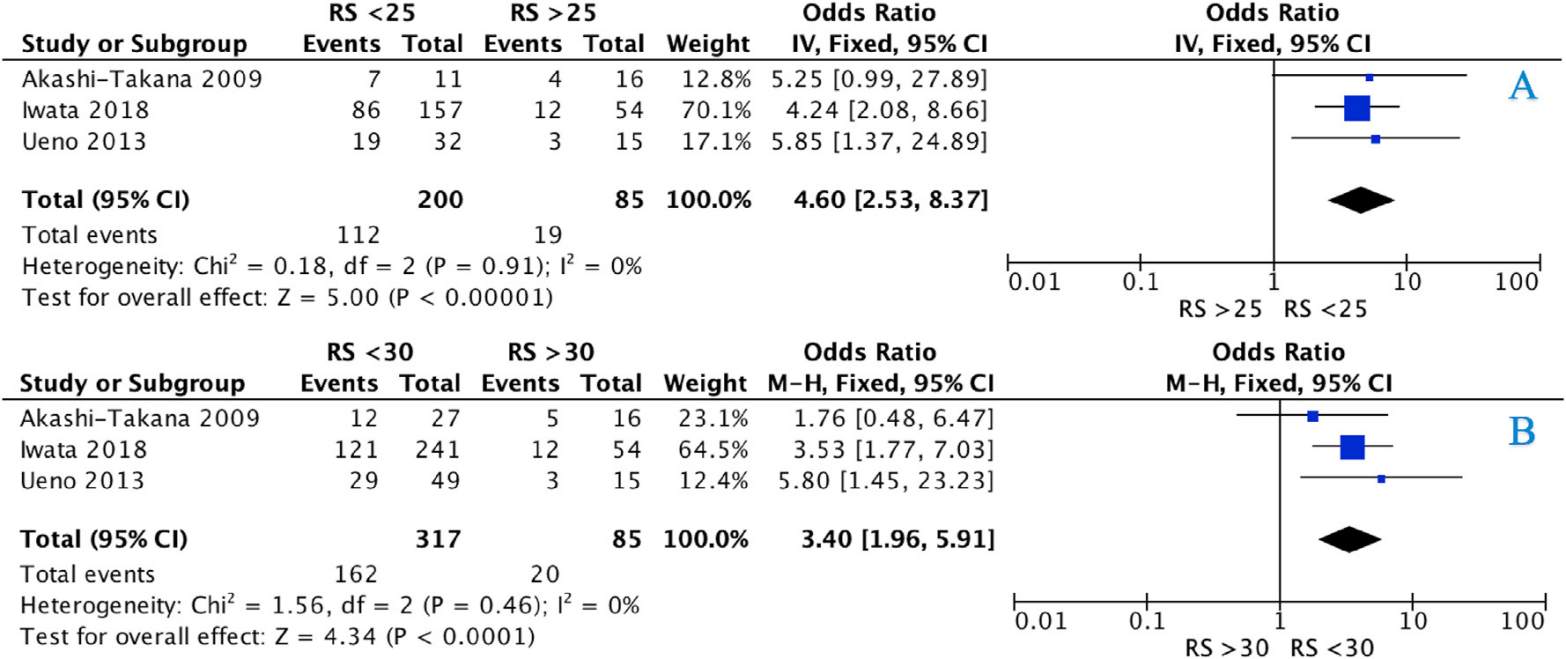
Forest plots illustrating response to neoadjuvant endocrine therapy in patients using OncotypeDX© Recurrence Score of 25 (Fig. 2A) and 30 (Fig. 2B) as respective clinical cut-off points on core tissue biopsy.

Only 22 patients achieved breast pCR (2.8%) and RS group was not associated with pCR (*P* = 0.850, Chi-Squared test, *χ*^*2*^). Only Bear et al. reported on axillary pCR (0.0%, 0/30) [20]. Seven studies reported on PR following NET. Patients with RS < 18 were associated with PR (*P* < 0.001, *χ*^*2*^) (Supplementary Appendix 2). Patients with RS < 25 (OR: 4.60, 95% CI: 2.53–8.37, *P* < 0.001, *I*^*2*^ = 0%) and those with RS < 30 (OR: 3.40, 95% CI: 1.96–5.91, *P* < 0.001, *I*^*2*^ = 0%) were more likely to achieve PR to NET (Fig. 2).

Four studies reported SD and DP following NET. RS group failed to predict SD (OR: 2.67, 95% CI: 0.34–21.27, *P* = 0.350, *I*^*2*^ = 87%) and failed to predict DP (OR: 0.20, 95% CI: 0.01–3.81, *P* = 0.280, *I*^*2*^ = 79%) following NET (Supplementary Appendices 3. A & 3. B).

### 3.6 Breast conservation surgery post neoadjuvant endocrine therapy

Three studies reported on achieving BCS following NET: Patients with RS < 18 were associated with BCS following NET (*P* < 0.001, †) (Table 3). However, for those with RS < 18 receiving NET failed to increase BCS conversion rates (OR: 0.23, 95% CI: 0.04–1.47, *P* = 0.120, *I*^*2*^ = 85%) (Supplementary Appendix 3.C). For patients with RS > 30, NET prescription was not associated with BCS (*P* = 0.469, †) (Table 3), and failed to increase BCS conversion rates for those with RS > 30 (OR: 1.27, 95% CI: 0.64–2.49, *P* = 0.490, *I*^*2*^ = 0%) (Supplementary Appendix 3.D).

**Table 3:** Rates of breast conservation surgery and 5-year disease free survival for each OncotypeDX© Recurrence Score group.

	RS < 18	RS 18-30	RS <30	RS >30	*P-*value
Pre-NET BCS	111/189	6/17	311/528	40/69	<0.001∗*χ*^*2*^
Post-NET BCS	147/189	13/17	0/12	30/69	0.469^a^
5-year DFS	39/42	25/32	–	18/28	0.012∗*χ*^*2*^

RS; OncotypeDX© Recurrence Score, NET; neoadjuvant endocrine therapy, BCS; breast conservation surgery, DFS; disease-free survival.

*χ*^*2*^ denotes Chi-Squared test.

a Denotes Fisher's Exact test.

### 3.7 Oncological outcomes post neoadjuvant endocrine therapy

**Fig. 3:**
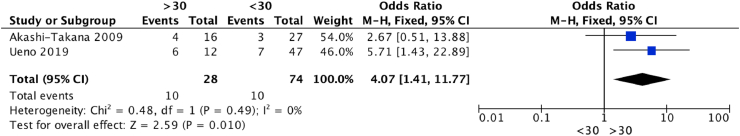
Forest plot illustrating the risk of disease recurrence 5-years following neoadjuvant endocrine therapy and surgical resection using OncotypeDX© Recurrence Score of 30 as the clinical cut-off.

Only two studies reported 5-year DFS outcomes following NET prescription: In these analyses, patients with RS < 18 had significantly less recurrence than those with RS > 18 (*P* = 0.012, *χ*^*2*^) (Table 3). Of those with RS > 18, 58.1% received adjuvant chemotherapy (36/62). Patients with RS > 30 suffered more disease recurrence than their counterparts (OR: 4.07, 95% CI: 1.41–11.77, *P* = 0.010, *I*^*2*^ = 0%) (Fig. 3).

## 4 Discussion

In early-stage breast cancer, therapeutic decision making has been personalised in ER+/HER2-disease through substratification by multigene panels, such as the 21-gene expression signature. The purpose of the current systematic review and meta-analysis was to assess the predictive value of RS results for response to NET in patients diagnosed with ER+/HER2-breast cancer on their core tissue biopsy, and the most important finding is data indicating patients with low or intermediate RS are estimated to be four times more likely to achieve a response to NET than those with high-risk RS. This analysis highlights the value of performing RS testing in the preoperative setting, as those with low-intermediate RS may be spared unnecessary overtreatment with NAC, while still achieving preoperative downstaging following 4–6-months of NET. While molecular profiling has now become embedded into the guidelines for guiding cytotoxic chemotherapy prescription in the adjuvant setting in the context of ER+/HER2-disease [43,44], the paradigm is evolving such that current efforts are focused upon expanding indications of RS testing into new settings, such as cases of increased nodal burden (as observed in the SWOG S1007 trial) [15], or into neoadjuvant practice [19]. Thus, the current analysis supports the novel application of the 21-gene assay into the preoperative setting to guide prescription of neoadjuvant endocrine agents, where indicated.

It has been well recognized in recent times that cancers expressing ER+/HER2-have poor sensitivity to systemic chemotherapy [17,18], with pCR rates following NAC rarely exceeding 10% [4,19]. In spite of this, NAC remains a therapeutic strategy of choice for patients with locally advanced (IIb-IIIc) disease, in those requiring pre-operative downstaging, and in women hoping for breast conservation despite an increased tumour to breast ratio [45]. In conventional ER + breast cancer management, NET is typically prescribed in 3.1% of cases, compared to NAC in 24.7% of cases [46]. In their meta-analysis of prospective trials, Spring et al. describe similar pCR rates for patients with ER + disease receiving NET *vs*. those prescribed NAC, however, suffer significantly less toxicities [47]. In the current study, a paucity of patients achieved breast pCR following NET, with a pCR rate of 2.8%. In their recent meta-analysis, Boland et al. illustrate ER+/HER-cancers with high-risk RS having increased rates of pCR compared to low-intermediate risk patients (9.5% *vs*. 2.1%) [19]. These results highlight comparable pCR rates following NET and NAC for patients with low-intermediate RS. Thus, given the reduced morbidity rates [47], data from this analysis suggest patients with low-intermediate RS would be best served with NET, or by proceeding directly to surgical resection. However, with respect to oncological outcome, patients with RS > 30 suffered increased rates of disease recurrence than their counterparts (OR: 4.07). Caution must be taken when interpreting data with regard to DFS; in this context, the effect of NET in DFS would likely be completely confounded and diluted by the prescription of combined adjuvant chemoendocrine therapies. In this setting, it seems RS is an accurate means of cancer prognostication and should therapeutic strategies should adhere to best practice guidelines [43,44].

Notwithstanding poor pCR rates, the current study suggests NET is an appropriate strategy in tumour downstaging and bridging to cancer surgery for patients with low-intermediate RS. In this analysis, cancers with RS < 30 were 3.4 times as likely to respond to NET, while those with RS < 25 estimated to be 4.6 times as likely to have a response. Furthermore, despite NET prescription failing to predict BCS rates patients with RS < 18 in our meta-analysis, we observed a 20% increase in BCS post-NET (77.8% vs. 58.7%), illustrating value for its use following substratification by the 21-gene assay. These results supplement extrapolated data from initial European and American studies outlining the value of 3–4 months of NET prescription in successfully facilitating tumour downstaging to breast conservation [[48], [49], [50]]. Following NET prescription, pCR seems a less achievable primary analytical endpoint when compared to NAC, building argument for inclusion of reduction of tumour cellularity, successful BCS, or molecular parameters, such as Ki-67 or Preoperative Endocrine Prognostic (PEPI) Indices to be incorporated into planning of prospective NET studies [50,51]. Dowsett et al. correlated the degree of Ki-67 suppression to recurrence-free survival following NET for those with ER+/HER2-disease [51], and Ellis et al. incorporated Ki-67 expression into their PEPI scoring signature, which is used to identify patients at low risk of relapse following NET, and whom may be spared adjuvant cytotoxic chemotherapy [50]. Given the aforementioned limitations of pCR, these clinical, surgical and molecular analytical endpoints may be favourable primary outcomes for prospective NET studies and analyses.

Despite these promising results, the authors wish to highlight a number of potential barriers surrounding the adoption of preoperative NET into clinical practice, including the heterogenous nature of individual responses, the long duration of treatment required to achieve a clinical response, as well as the possibility that primary surgery may be favourable for a majority of patients [52]. However, in the wake of the COVID-19 pandemic, breast cancer surgery has had to adapt practice to minimize potential exposure to COVID-19, while ensuring patient oncological safety [53]. Based on tumour stage and molecular biology, professional bodies have provided recommendations to identifying those for whom surgery is ‘time-critical’, and patients where surgical management may be deferred [54]. As a consequence, NET prescription has been implemented to ‘bridge’ patients to surgery in cases of ER+, early-stage disease [55]. Therefore, the data presented in the current study advocates for neoadjuvant RS testing to facilitate appropriate NET prescription for those with deferred surgery during these challenging times in the world of oncology.

In the current study, patients treated with NET in the RS > 30 group were 4 times more likely to suffer a disease recurrence at 5-years follow-up than their counterparts. This proves somewhat unsurprising: Seminal work from Paik et al. had previously illustrated inferior survival outcomes for those with increased RS, in particular in the absence of combined chemoendocrine therapy [13,14]. Paik determined Kaplan-Meier estimates of distant recurrence rates at 10-years to be 6.8%, 14.3% and 30.5% for patients with traditional low-, intermediate- and high-risk RS. In contrast to Paik et al. the current analysis provides poorer disease control after just 5-years follow-up, with recurrence rates of 7.1%, 21.9% and 35.7% respectively when using traditional RS cutoffs. Although this may cause concern in relation to NET prescription indicated through RS testing, the authors wish to highlight certain discrepancies within included patient characteristics. In our analysis, patients with T3/T4 and N1/N2 disease were included. All patients analysed by Paik had T1/T2 cancers, with only 5% exceeding 40 mm in size, and none having disease in the axilla. Traditionally, tumour stage and degree of nodal involvement are well recognized as biomarkers predictive of clinical outcomes in breast cancer, with large European cohorts validating this concept following the advent of Nottingham Prognostic Index (NPI) by Haybittle et al., in 1982 [56,57]. Therefore, while best efforts remain in personalizing oncological practice through genomic profiling, recognition of tumour burden as an indicator of prognosis is crucial within each molecular subtype, limiting conclusions which can be drawn in relation to survival in this analysis.

### 4.1 Limitations

This study utilizes pCR as a primary analytical endpoint, the validity of which may be questioned in the context of NET prescription. Future prospective analyses evaluating the utility of NET in ER+/HER2-disease may concentrate on the absolute reduction in tumour cellularity, drop in Ki-67 expression or PEPI scores. Included studies varied with the cut-offs used for differentiating RS groups, with recent data from Sparano et al. indicating that RS 25–30 should not be treated with endocrine agents in monotherapy and require combined chemoendocrine therapy in order to achieve survival benefit [17]. Our results suggest low-intermediate recurrence score achieve a response to NET, however definitions in response vary between studies, with some included studies failing to clearly define and indicate adequate response. Overall, BCS conversion rates may be considered a subjective parameter, with inter-surgeon variability known to impact surgical decision making in breast cancer surgery [58]. Due to few studies being published assessing the value NET following RS testing, authors included three studies from which data was retrieved from abstracts, as full-text manuscripts are currently not published. While this analysis suggests that NET prescription following RS may be reasonable in tumour downstaging, 90% of cancers had T1/T2 disease limiting conclusions which may be drawn in relation to downstaging T3 or locally advanced disease. Recent data from Bernhardt et al. suggests there may be discrepancies between RS values on core biopsy and resected tumour specimens, albeit impacted by patient age [59]. Patient data from two separate cohorts (both Ueno et al.) [41,42] from the JFMC34-0601 multi-centre prospective study (N = 107) were included despite uncertainty existing in relation to some potentially overlapping patients. Finally, this analysis incorporates data from less than 700 patients (including one small analysis of just 15 patients) who have undergone NET following RS testing on diagnostic core biopsies; larger, prospective studies are required to add to the current literature surrounding the clinical utility of the 21-gene assay in guiding therapeutic decision making in the neoadjuvant setting.

In conclusion, the current systematic review and meta-analysis advocates for NET prescription to be considered in cases where tumour downstaging may be required preoperatively and where genomic substratification by the 21-gene assay has been performed on core needle biopsy diagnostic tissue. Results from this analysis indicate that those with low- or intermediate-risk RS on core biopsy are four times more likely to respond to NET than those with high-risk RS and recent evidence demonstrates that this same cohort demonstrates relative chemoresistance to NAC. This suggests that RS testing may be useful when performed on diagnostic biopsy in order to guide neoadjuvant therapies in ER+/HER2-breast cancer, with NET prescription a reasonable option to potentially downstage ER + cancers while providing data in relation to the in-vivo sensitivity of the tumour to endocrine agents for use in the adjuvant setting.
