# Supplementary material for: Clinical utility of the 21-gene assay in predicting response to neoadjuvant endocrine therapy in breast cancer: A systematic review and meta-analysis
Source: Breast. 2021 May 12;58:113–20. doi: 10.1016/j.breast.2021.04.010 (PMC8142274; doi:10.1016/j.breast.2021.04.010)
Supplement: Multimedia component 1 [file mmc1.docx]

**Clinical Utility of the 21-gene assay in Predicting Response to Neoadjuvant Endocrine Therapy in Breast Cancer: A Systematic Review and Meta-Analysis**

M.G. Davey MCh MRCS, É.J. Ryan MD MRCS, M.R. Boland MCh FRCS, M. K. Barry FRCS FACS, A.J. Lowery PhD FRCS, M.J. Kerin MCh FRCS FRCSI FRCSEd

The Lambe Institute for Translational Research, National University of Ireland, Galway, Ireland

Supplementary Appendix 1.

**Supplementary Appendix 1.** Clinicopathological characteristics of patients included in this analysis

| **Characteristic** | | **Abu-Khalaf** | **Akashi-Tanaka** | **Al-Saleh** | **Bear** | **Iwata** | **Khan** | **Ueno (2013)** | **Ueno (2019)** |
| --- | --- | --- | --- | --- | --- | --- | --- | --- | --- |
| **Age (years)** | (Range) | - | 61 (48-79) | (25-84) | 64 (41-80) | 63 (49-75) | 62.3 | 55-79 | 55-79 |
| **Menopausal status** | Premenopausal  Post-menopausal | - 15 | -  - | 135 103 | 3  28 | -  295 | -  - | -  - | -  59 |
| **Size (mm)** | - | - | - | - | 35 | 25 | 35 | - | - |
| **Tumour stage** | T1  T2  T3/T4 | -  -  - | -  27  16 | -  -  - | -  30  1 | 44  251  - | -  -  - | -  62  2 | -  57  2 |
| **Nodal stage** | N0  N1  N2 | -  -  - | 12  14  15 | -  -  - | 24  8  1 | -  -  - | - 6 - | -  -  - | 43  16  - |
| **Grade** | Grade 1  Grade 2  Grade 3 | -  -  - | -  -  - | -  -  - | 10  19  2 | 195  59  27 | 9  31  2 | -  -  - | -  -  - |
| **ER status** | ER positive  ER negative | 15  0 | 43  - | 238  - | 31  - | 293  2 | 42  - | 63  1 | 59  0 |
| **PgR status** | PgR positive  PgR negative | -  - | -  - | 238  - | 28  - | 211  84 | -  - | 50  14 | 55  4 |
| **HER2 status** | HER2 positive  HER2 equivocal  HER2 negative | 0  0  15 | -  -  - | -  -  - | -  -  - | 9  51  235 | -  -  - | 2  12  50 | 0  0  59 |
| **Ki-67 indices** | Ki67 0-10%  Ki67 10-30%  Ki67 >30% | -  -  - | -  -  - | -  -  - | -  -  - | 86  123  61 | -  -  - | 28  23  13 | -  -  - |

mm; millimetres, T; tumour stage, N; nodal stage, ER; estrogen receptor, PgR; progesterone receptor, HER2; human epidermal growth factor receptor-2, RS; OncotypeDX© Recurrence Score
