# Supplementary material for: Clinical utility of the 21-gene assay in predicting response to neoadjuvant endocrine therapy in breast cancer: A systematic review and meta-analysis
Source: Breast. 2021 May 12;58:113–20. doi: 10.1016/j.breast.2021.04.010 (PMC8142274; doi:10.1016/j.breast.2021.04.010)
Supplement: Multimedia component 2 [file mmc2.docx]

**Clinical Utility of the 21-gene assay in Predicting Response to Neoadjuvant Endocrine Therapy in Breast Cancer: A Systematic Review and Meta-Analysis**

M.G. Davey MCh MRCS, É.J. Ryan MD MRCS, M.R. Boland MCh FRCS, M. K. Barry FRCS FACS, A.J. Lowery PhD FRCS, M.J. Kerin MCh FRCS FRCSI FRCSEd

The Lambe Institute for Translational Research, National University of Ireland, Galway, Ireland

Supplementary Appendix 2.

**Supplementary Appendix 2**. Rates of response to neoadjuvant endocrine therapy for each OncotypeDX© Recurrence Score group.

|  | **RS <11** | **RS 11-25** | **RS <25** | **RS >25** | ***P-*value** | **RS <18** | **RS 18-30** | **RS <30** | **RS >30** | ***P-*value** |
| --- | --- | --- | --- | --- | --- | --- | --- | --- | --- | --- |
| pCR | 1/13 | 4/18 | 4/57 | - | 0.225 † | 0/32 | 0/17 | 0/12 | 9/287 | 0.850 *χ^2^,* 0.990† |
| PR | 10/13 | 9/18 | 39/57 | - | 0.252 † | 112/200 | 50/117 | 311/528 | 20/85 | <0.001* *χ^2^,* † |
| SD | - | - | - | 22/27 | - | 82/189 | 53/101 | 142/528 | 39/290 | <0.001* *χ^2^,* † |
| DP | - | - | 1/42 | - | - | 1/157 | 3/84 | 8/317 | 14/69 | <0.001* *χ^2^,* † |

RS; OncotypeDX© Recurrence Score, PR; partial response, pCR; pathological complete response, SD; stable disease, DP; disease progression

† denotes Fisher’s Exact test

*χ^2^* denotes Chi-Squared test
