# Supplementary material for: Clinical utility of the 21-gene assay in predicting response to neoadjuvant endocrine therapy in breast cancer: A systematic review and meta-analysis
Source: Breast. 2021 May 12;58:113–20. doi: 10.1016/j.breast.2021.04.010 (PMC8142274; doi:10.1016/j.breast.2021.04.010)
Supplement: Multimedia component 3 [file mmc3.docx]

**Clinical Utility of the 21-gene assay in Predicting Response to Neoadjuvant Endocrine Therapy in Breast Cancer: A Systematic Review and Meta-Analysis**

M.G. Davey MCh MRCS, É.J. Ryan MD MRCS, M.R. Boland MCh FRCS, M. K. Barry FRCS FACS, A.J. Lowery PhD FRCS, M.J. Kerin MCh FRCS FRCSI FRCSEd

The Lambe Institute for Translational Research, National University of Ireland, Galway, Ireland

Supplementary Appendix 3.


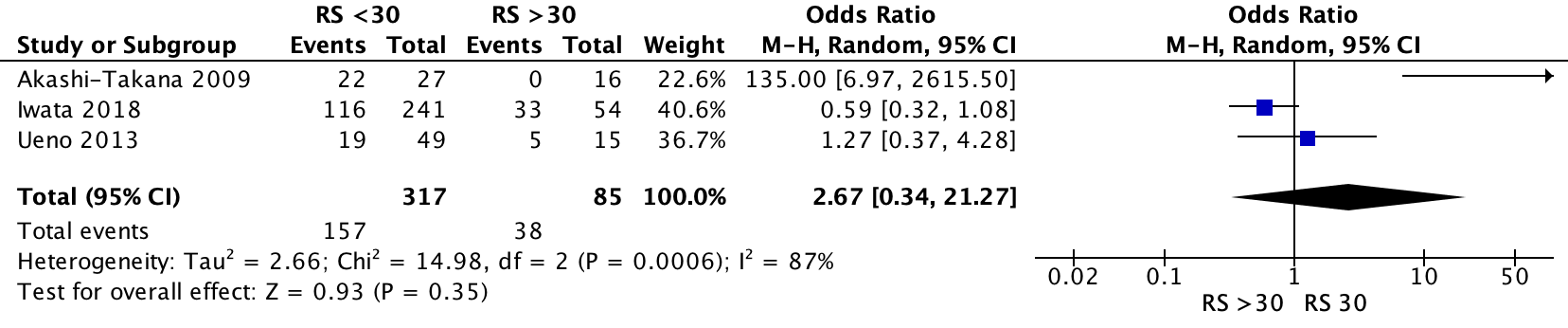

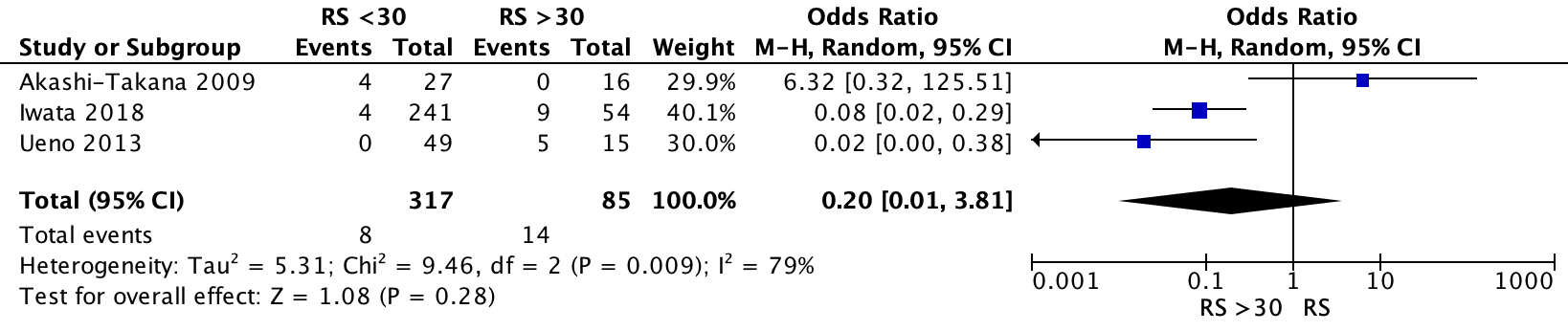

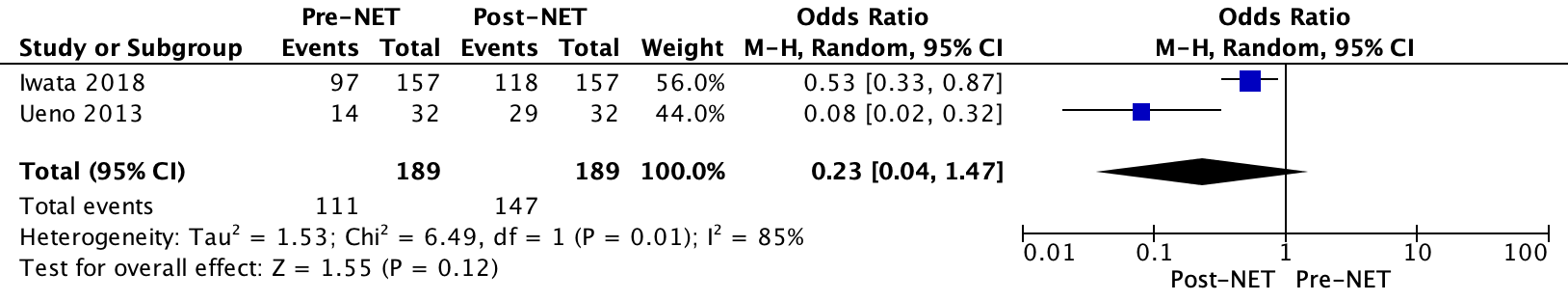

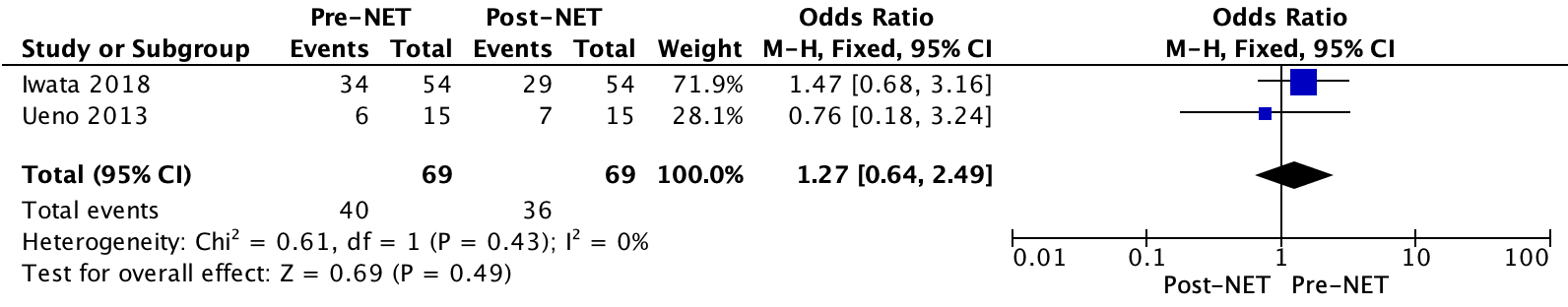


D

C

A

B

**Supplementary Appendix 3**.

Forest plots illustrating the likelihood of having (A) stable disease and (B) disease progression following neoadjuvant endocrine therapy (NET) for those with OncotypeDX© Recurrence Score (RS) <30 *vs.* RS >30. Forest plots C and D illustrate the rates of breast conservation surgery in those receiving NET using (C) RS of 18 and (D) 30 as respective clinical cut-off points on core tissue biopsy.
